# PNIPAM-MAPOSS Hybrid Hydrogels with Excellent Swelling Behavior and Enhanced Mechanical Performance: Preparation and Drug Release of 5-Fluorouracil

**DOI:** 10.3390/polym10020137

**Published:** 2018-01-31

**Authors:** Peihong Li, Xiaoman Hou, Lijie Qu, Xueyan Dai, Chunling Zhang

**Affiliations:** Key Laboratory of Automobile Materials, Ministry of Education, College of Materials Science and Engineering, Jilin University, Changchun 130022, China; phli16@mails.jlu.edu.cn (P.L.); houxm1205@163.com (X.H.); qulijie92528@outlook.com (L.Q.); godjealouselite@163.com (X.D.)

**Keywords:** hybrid hydrogel, MAPOSS, mechanical properties, swelling, drug release

## Abstract

Poly(*N*-isopropylacrylamide) (PNIPAM) is a widely-studied polymers due to its excellent temperature sensitivity. PNIPAM-MAPOSS hybrid hydrogel, based on the introduction of acrylolsobutyl polyhedral oligomeric silsesquioxane (MAPOSS) into the PNIPAM matrix in the presence of polyethylene glycol, was prepared via radical polymerization. The modified hydrogels exhibited a thick, heterogeneous porous structure. PEG was used as a pore-forming agent to adjust the pore size. MAPOSS reduced the swelling ratios of gels, and decreased the LCST, causing the hydrogels to shrink at lower temperatures. However, its hydrophobicity helped to improve the temperature response rate. The incorporation of rigid MAPOSS into the polymer network greatly increased the compressive modulus of the hydrogel. It is worth noting that, by adjusting the amount of MAPOSS and PEG, the hydrogel could have both ideal mechanical properties and swelling behavior. In addition, hydrogel containing 8.33 wt % MAPOSS could achieve stable and sustained drug release. Thus, the prepared PNIPAM-MAPOSS hybrid hydrogel can serve as drug carrier for 5-fluorouracil and may have potential application in other biomedical fields.

## 1. Introduction

Intelligent hydrogels have attracted considerable attention because of their abilities to change its volume and property in response to external stimuli, such as temperature [1], pH [2], light [3], ionic strength [4], electric fields [5], and magnetic fields [6]. Among all these stimuli-sensitive hydrogels, poly(*N*-isopropylacrylamide) (PNIPAM) is one of the widely-studied polymers due to its excellent temperature sensitivity. A series of studieshave been made to research its potential application, such as dye absorption [7,8], tissue engineering [9], and drug delivery [10,11,12]. The PNIPAM-based hydrogels undergo an abrupt volume phase transition at a lower critical solution temperature (LCST) of around 32 °C [13]. During the drug delivery, PNIPAM hydrogels swell and load the drug at a temperature below LCST, and shrink and release the loaded drug when the temperature rises above LCST. Typical types of polymerization are free radical polymerization and reversible addition-fragmentation chain transfer polymerization (RAFT).Polymerization methods include solution polymerization, inverse microemulsion polymerization [14], inverse suspension polymerization [15], precipitation polymerization [16], etc.

However, the slow temperature response rate and the low mechanical strength are common disadvantages of hydrogels. There are many general methods to modify hydrogels, including cross-linking, nanocomposites [17], porous structures [18], IPNs [19,20], etc. Polyhedral oligomeric silsesquioxane (POSS) with a cubic cage-like nanostructure is emerging as a promising organic-inorganic nanoparticle for the creation of nanocomposites. It serves as a terminal or cross-linking curing center to meet the different needs of modified polymers. POSS-polymer nanocomposite combination property is excellent. The main functions of POSS are as follows: (1) to improve the thermal performance, thereby increasing the operating temperature of composite materials can withstand; (2) to strengthen the mechanical properties, which is mainly due to the rigid structure of POSS; (3) to improve the processing performance; (4) to improve flame retardancy, make the composite material have a significant delayed combustion characteristics; and (5) to make it multi-functional. POSS modified hydrogels generally have good mechanical properties, response rate and thermal stability [21,22,23,24,25]. There are many kinds of such nanoparticles. The MAPOSS used in this paper carries carbon-carbon double bond functional groups. Therefore, it can be introduced into the matrix by cross-linking.

We have synthesized a series of MAPOSS-incorporated P(NIPAM-*co*-PEGDA) hybrid hydrogels in previous work [26].The introduction of POSS can improve some properties of hydrogels, especially mechanical properties, but this also weakens the water retention. In this paper, PNIPAM was still used as a matrix, a small amount of PEGDA as a crosslinking agent. We prepared a PNIPAM-MAPOSS hybrid hydrogel, and introduced a pore-forming agent PEG to adjust the pore structure. We focused on the swelling behavior and mechanical properties of this hydrogel. Then we studied its controlled release of the anti-cancer drug 5-fluorouracil.The results show that, the prepared gels had temperature sensitivity, excellent swelling behavior, enhanced mechanical properties, and potential application in drug delivery system.

## 2. Experimental Section

### 2.1. Materials

Acrylolsobutyl polyhedral oligomeric silsesquioxane (MAPOSS) was purchased from Hybrid Plastics Co. (Hattiesburg, MS, USA). *N*-isopropylacrylamide (NIPAM), polyethylene glycol diacrylate (PEGDA, average molecular weight ~200) and 5-fluorouracil (5-FU) were Aladdin (Shanghai, China) products. Polyethylene glycol (PEG 4000), 2,2′-azobis(2-methylpropionitrile) (AIBN), and tetrahydrofuran (THF) were Sinopharm (Shanghai, China) products. PEG 4000 and AIBN are chemical pure and THF is analytically pure. All reagents were used as received. Phosphate buffer solution (PBS, pH 7.4) was prepared using potassium dihydrogen phosphate (1.36 g) and NaOH solution (0.1 mol/L, 79 mL), followed by diluting the solution to 200 mL with ultrapure water.

### 2.2. Preparation of PNIPAM-MAPOSS Hybrid Hydrogels

**Figure 1:**
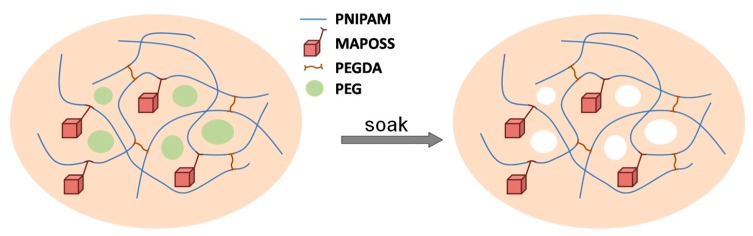
Schematic of preparation of PNIPAM-MAPOSS hybrid hydrogels.

All types of PNIPAM-MAPOSS hybrid hydrogels were prepared by one-step radical polymerization. The synthesis process can refer to our previous work [26].Briefly, 1 g NIPAM and 0.10 g PEGDA with different proportions of MAPOSS and PEG were dissolved in THF at room temperature. Then 0.01 g AIBN was added to the mixture dispersion with stirring. The resulting solution was transferred to a glass tube and placed in a water bath at 60 °C. All samples were taken out after the reaction, and immersed in THF and deionized water alternately for threedays to remove PEG and impurities. A schematic is shown in Figure 1. The detailed feed ratios of each group of PNIPAM-MAPOSS hybrid hydrogels are listed in Table 1.

### 2.3. Characterization

Fourier-transform infrared spectrometry (FTIR) spectra of hydrogels were recorded using KBr disks on a Nicolet Nexus 670 spectrometer (Nicolet, Madison, WI, USA). In all case, the scans were carried out in the spectral range varying from 4000 to 400 cm^−1^ with a resolution of 4 cm^−1^. The interior morphologies of the freeze dried specimens were observed by environmental scanning electron microscopy (ESEM; XL30 ESEM-FEG, FEI, Hillsboro, OR, USA) after sputter-coating with gold under vacuum. The LCST measurement of the wet samples from 20 to 50 °C was determined by differential scanning calorimetry (DSC; Q20, TA, New Castle, PA, USA) under a nitrogen atmosphere with a heating rate of 2 °C/min.

### 2.4. Swelling Equilibrium of the PNIPAM-MAPOSS Hybrid Hydrogels

The pre-weighed dry hydrogels were immersed in deionized water at 20 to 50 °C until swelling equilibrium was reached. The weight of hydrogel was measured after removing the excess water on the surface with wet filter paper. The swelling ratio (*SR*) was determined by the gravimetric method and calculated by following Equation (1):

(1) $$SR=\frac{W_{s}-W_{d}}{W_{d}},$$

where *W*_s_ is the weight of the swollen hydrogel reached swelling equilibrium at a given temperature, and *W*_d_ is the weight of dry hydrogel [27].

The deswelling behavior was investigated by recording the change in the weight of water in the hydrogels over time. The hydrogels samples first reached swelling equilibrium at 25 °C, and were then placed in water at 60 °C. The samples were recorded at regular time intervals. Water retention (*WR*) corresponding to the deswelling ratio is defined by Equation (3):

(2) $$WR=\frac{W_{f}-W_{d}}{W_{s}-W_{d}}\times 100\%,$$

where *W*_f_ is the weight of the hydrogel at a certain time, *W*_s_ is the ultimate weight of the swollen hydrogel reached swelling equilibrium at 25 °C, and *W*_d_ is the weight of dry hydrogel [28]. Five independent samples were tested and plotted with the average of their *SR*_s_ and *WR*_s_.

### 2.5. Mechanical Performance Test

The mechanical properties of PNIPAM-MAPOSS hybrid hydrogels were investigated with a WSM universal mechanical tester (Changchun Intelligent Equipment Co., Ltd., Changchun, China) at room temperature. The cylindrical samples (10 mm in diameter, 10 mm in height) were immersed in deionized water and kept at 25 °C for at least 24 h prior to testing.

Since the hydrogels are soft materials, all the samples were compressed at a rate of 10 mm/min until the compression ratio reached 70%. According to the obtained stress-strain curves, the hydrogel compressive tangent modulus was calculated by the finite difference method at 5% and 20% strain. The equation is follows:

(3) $$E_{\varepsilon }=\frac{\sigma_{\varepsilon +\Delta \varepsilon }-\sigma_{\varepsilon -\Delta \varepsilon }}{2\Delta \varepsilon },$$

Where *E*_ε_ is the compressive tangent modulus of the hydrogel at the compressive strain ratio of ε. Δε is the variable of the strain, taking 2% [29]. In addition, five independent samples were tested and the average was calculated.

### 2.6. Drug Release Behavior of 5-Fluorouracil

The drug-loaded hydrogels were prepared by using 5-FU as a model drug. The disc-like dried hydrogel was immersed in 5-FU solution (1 mg/mL) at 25 °C for 48 h to reach swelling equilibrium, and then dried using vacuum freezing to obtain a drug-loaded hydrogel. The in vitro release behavior of 5-FU from PNIPAM-MAPOSS hybrid hydrogels was studied in PBS (pH 7.4) at physiological temperature. The PBS was shocked at 100 rpm. At each fixed time interval, 3 mL solution was taken out and replaced by 3 mL of fresh PBS to maintain the volume of the medium. There are five independent samples for each kind of gel for testing. The released amount of 5-FU was measured by ultraviolet spectrophotometer (UV; UV-6100s, MAPADA, Shanghai, China) at 265 nm [30]. The average of released amounts was calculated for each group.

## 3. Results and Discussion

### 3.1. FTIR Spectroscopy

**Figure 2:**
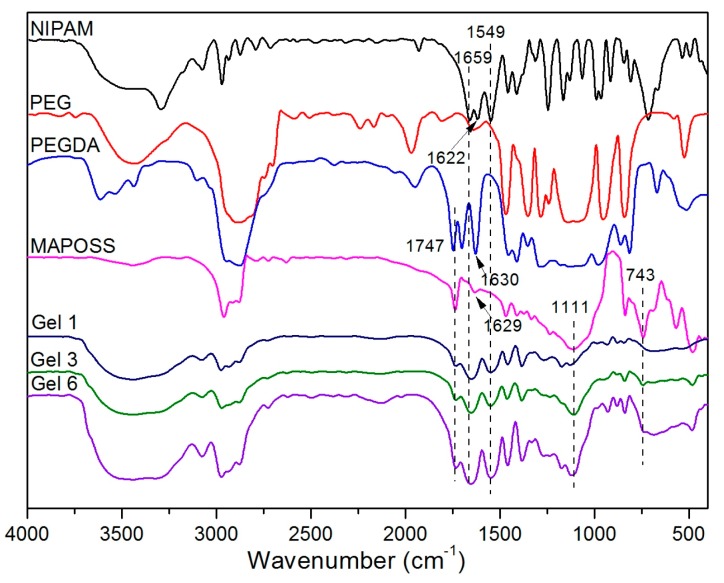
FTIR spectra of NIPAM, PEG, PEGDA, MAPOSS, and PNIPAM-MAPOSS hybrid hydrogels.

The FTIR spectra of PNIPAM-MAPOSS hybrid hydrogels are shown in Figure 2. The characteristic peaks of NIPAM at 1659 and 1549 cm^−1^ are assigned to the C=O stretching vibration and N–H deformation vibration of amide group. The typical peak appearing at 1747 cm^−1^ belongs to the C=O stretching vibration of ester group in PEGDA and MAPOSS. The peaks at 1622, 1630, and 1629 cm^−1^, related to the C=C stretching vibrations in NIPAM, PEGDA and MAPOSS macromer, disappeared in PNIPAM-MAPOSS hybrid hydrogels (Gel 1, 3, 6), indicating chemical cross-linking between the three. The peaks of Si–O–Si and Si–C of MAPOSS at 1111 and 743 cm^−1^ could be found in Gel 3 and 6, indicating the successful incorporation of MAPOSS into Gel 3 and 6, but not Gel 1. It should be pointed out that, the absence of terminal hydroxyl at 3445 cm^−1^ in hybrid hydrogels confirmed the remove of PEG. PEG only acted as a pore-forming agent and did not participate in the reaction.

### 3.2. Interior Morphology

**Figure 3:**
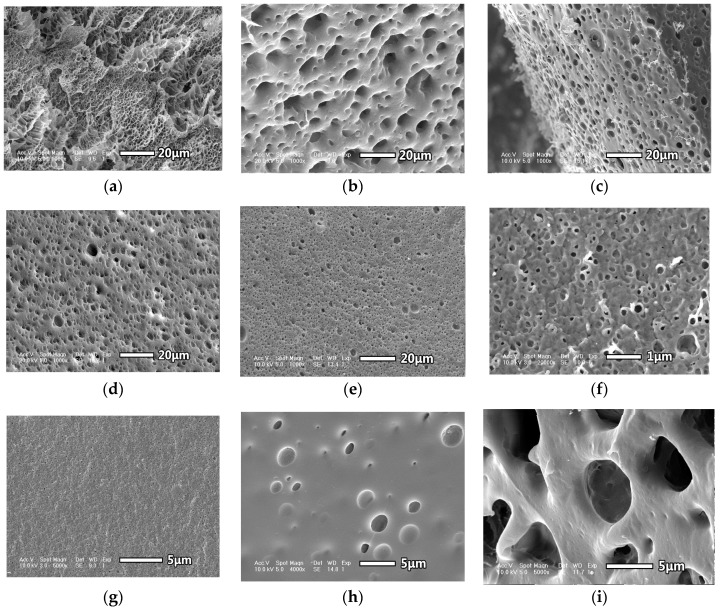
SEM images of (**a**) Gel 1; (**b**) Gel 2; (**c**) Gel 3; (**d**) Gel 4; (**e**) Gel 5; (**f**,**g**) Gel 6; (**h**) Gel 7; and (**i**) Gel 8.

The SEM images were used to study the effect of MAPOSS and PEG on the porous structure of gels. Figure 3 shows the interior pore of these hydrogels. Gel 1 without MAPOSS exhibited a porous morphology with a very thin wall, which is shown in detail in Figure 3a. The pores of Gel 2 became larger and heterogeneous than Gel 1 due to the introduction of 4.35 wt % MAPOSS. It can be directly observed from the SEM images that the pore size decreased as the MAPOSS content increased (average pore size: Gel 2 > Gel 3 > Gel 4 > Gel 5). The distinct cubic core (0.53 nm) and the functionalized arms of MAPOSS occupied some space in the network structure. Therefore, the pore walls of the hybrid hydrogels became thicker and their structural homogeneity deteriorated. The formation of a heterogeneous, porous structure in hydrogel is usually ascribed to polymerization-induced microphase-separation. MAPOSS and hydrophobic portions of PNIPAM self-assembled into hydrophobic domains. These hydrophobic MAPOSS domains would also act as cross-linking sites connecting PNIPAM chains, so that the increase of MAPOSS made a more compact network structure, leading the pore size reduced. On the contrary, it is evident that the pore diameter of Gel 6 without PEG was only 200 nm, while the Gel 8 exhibited macroporous structure with pore diameter of 3 to 7 μm (see Figure S1). The amount of pore-forming agent PEG was directly related to the pore size (average pore size: Gel 8 > Gel 3 > Gel 7 > Gel 6). After the polymerization, we soaked gels in THF and deionized water alternately to remove PEG. The original position occupied by PEG became a pore, and these pores were regular in size.

### 3.3. Volume Phase Transition Behavior

**Figure 4:**
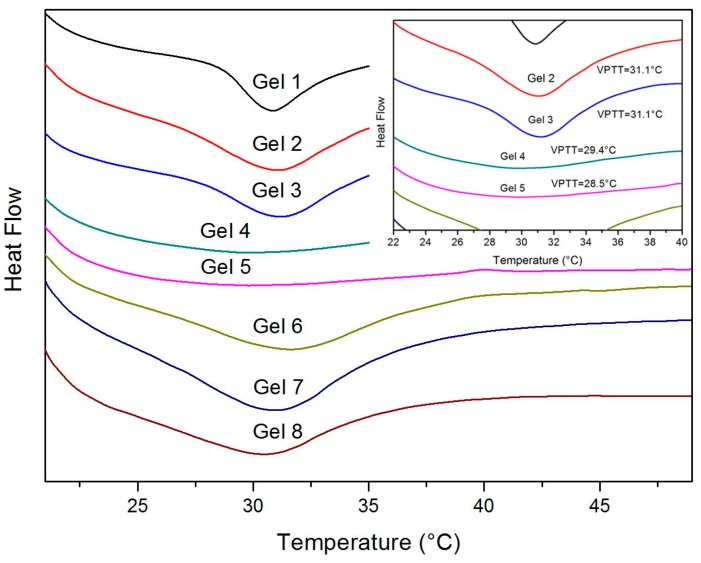
The phase transition behavior of gels measured by DSC.

PNIPAM-based hydrogels are temperature sensitive. In essence, the volume change of the temperature-sensitive hydrogel in the sense of temperature changes is a phase separation process, so that the phase separation heat is accompanied. The DSC thermograms of hydrogels are shown in Figure 4, and the LCSTs can be found in Table 1. We defined the endothermic peak on the curve as the LCST.

The results revealed that, when the amount of MAPOSS increased from 0 to 21.43 wt %, the LCSTs decreased from 31.1 to 28.5 °C. PNIPAM has inherently hydrophilic segments and hydrophobic segments, but this hydrophilic-hydrophobic balance of hydrogel was changed by adding MAPOSS. With more hydrophobic components, the affinity of the gels with water reduced and the LCST decreased. Even the endothermic peaks of Gel 4 and Gel 5 became insignificant. Too much MAPOSS weakened the temperature sensitivity of hydrogels. Hence, PNIPAM-MAPOSS hybrid hydrogels could shrink at a lower temperature. In addition, the use of PEG to prepare hydrogels had little impact on its LCST.

### 3.4. Swelling and Deswelling Behavior

**Figure 5:**
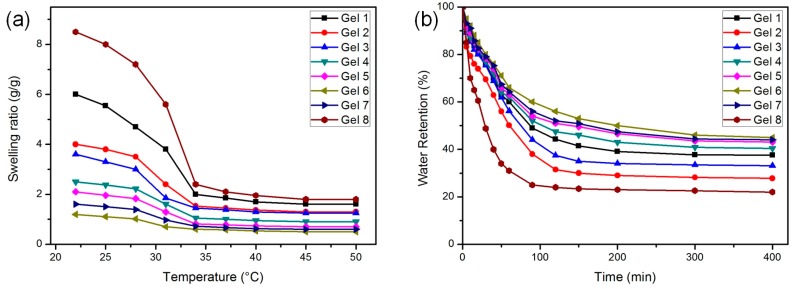
(**a**) The temperature-dependent swelling ratios of gels between 20 and 50 °C; and (**b**) deswelling behavior of gels at 60 °C.

The swelling behavior was used to study the temperature sensitivity of gels, while the deswelling behavior was used to study the response rate. The temperature-dependent swelling ratios (*SR*s) of hydrogels were investigated at different temperatures, as shown in Figure 5a. All samples had the volume phase transition behavior and exhibited excellent thermal responsiveness, supported by their *SR*s reduction, especially between 22.0 and 34.0 °C. At temperatures above LCST, the *SR*s were relatively small, but not very different, because the hydrogels shrunk at high temperatures and the network structure became similar.

MAPOSS was not conducive to swelling. From the SEM images, we already know that the MAPOSS caused the internal pores of the hydrogel smaller and heterogeneous. Both the tight network structure and hydrophobicity reduced the *SR*s of gels. However, it was intuitively observed in Figure 5a that the addition of hydrophobic MAPOSS allowed the hydrogels to shrink at lower temperatures. As shown in Figure 5b, the relationship between the deswelling rate and the MAPOSS content was varied. Gel 2 reached equilibrium at 180 min, which deswelling rate was faster than Gel 1 because of the incorporation of MAPOSS. The hydrophobic domains could behave as the micro-porogens and, therefore, the contact area between polymer chains and water molecules were significantly increased. This would facilitate the diffusion of water molecules in the cross-linked networks. However, the deswelling rate became slower when the content of MAPOSS reached 15.38 wt % (Gel 4). After analysis, too much MAPOSS made the internal pores of hydrogel very small to limit water loss.

The introduction of PEG could significantly change the size of the pores inside the gels, thereby affecting the swelling behavior. The *SR* of Gel 8 (with the highest PEG content) was apparently higher than others. Larger internal pores could accommodate more water, so that the more the amount of PEG used, the higher the gel’s *SR*. Gel 8 also exhibited the fastest deswelling rate in Figure 5b. Gel 8 lost more than 75% water within 90 min, while only 40% water was evacuated from Gel 6. All the comparative tests showed that, the hydrogel with big internal pore had a large swelling ratio, fast deswelling rate, but poor water retention capacity. In summary, the temperature response behavior of the hydrogel was not only related to the structure of the pores, but also to the hydrophobicity of the substance. Hybrid hydrogel with a suitable swelling ratio and response rate could be prepared by co-adjusting the amounts of MAPOSS and PEG.

### 3.5. Mechanical Performance

**Figure 6:**
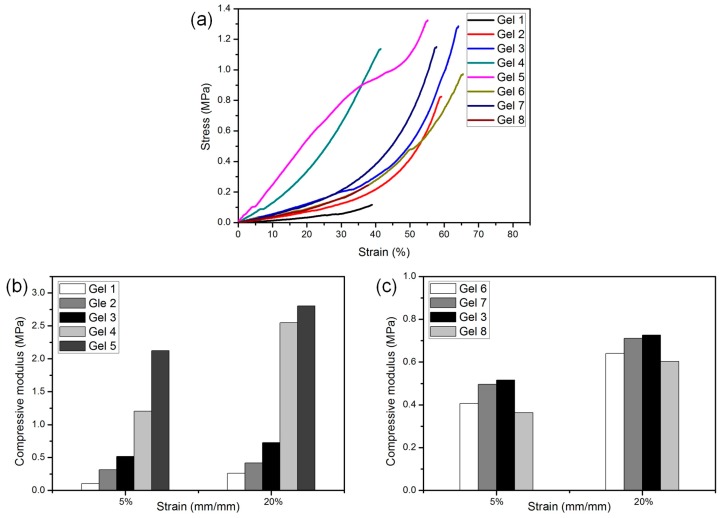
(**a**) Compression performance of gels: compression stress-strain curves; and (**b**,**c**) compressive tangent modulus at 5% strain and 20% strain.

The compression stress–strain curves of gels were shown in Figure 6a. Figure 6b,c show the effect of MAPOSS and PEG on the compressive modulus of hydrogels, respectively. It was shown that the compressive tangent modulus (*E*_ε_) calculated at 5% and 20% strain followed the same tend. In Figure 6b, Gel 1 exhibited a poor compressive performance (only 0.1 MPa at 5% strain, 0.3 MPa at 20% strain), while the *E*_ε_ of Gel 5 had been dramatically increased to approximately 10 times (about 2.1 MPa at 5% strain, and 2.8 MPa at 20% strain). Only by adding 8.33 wt % MAPOSS in the hydrogels could make the *E*_ε_ of Gel 3 reach 0.5 MPa at 5% strain and 0.7 MPa at 20% strain. The increase of compressive modulus could be explained mainly by three reasons. Firstly, the incorporation of rigid inorganic POSS core into the hydrogel networks enhanced the hardness of polymer chains. Secondly, MAPOSS with double bonds played the role of a cross-linking agent. It increased the cross-linking degree of gels and made the network more robust. Finally, the dispersion of POSS cages at the nanometer level could significantly restrict the motions of macromolecular chains as shown in other POSS-reinforced polymers. MAPOSS was easy to cause polymers chains entanglement, also helped to improve the mechanical properties of hydrogels.

As shown in Figure 6c, with the increase of PEG content, the *E*_ε_ increased first and then decreased. Appropriate water absorption could better maintain the network structure of hydrogel. Hence, it had good mechanical properties. However, larger pore size and higher *SR* caused by too much PEG were counterproductive. However, the effect of PEG on the mechanical properties of gels was not as obvious as that of MAPOSS. The improved hydrogels were more stable and could withstand a certain pressure without being destroyed.

### 3.6. Drug Release of 5-Fluorouracil

**Figure 7:**
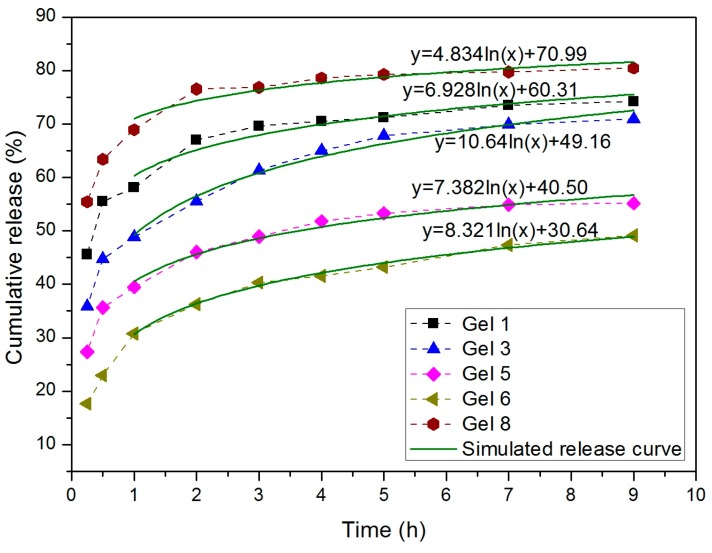
The release behaviors of 5-FU from gels in PBS (pH 7.4) at 37 °C and the simulated release curves.

5-Fluorouracil (5-FU) is an early anti-cancer drug and the most widely used in clinical anti-pyrimidine drugs [6]. It has a good therapeutic effect on gastrointestinal cancer and other solid tumors. However, its rapid metabolism in body may reduce the therapeutic effect [31]. Hsiue et al. synthesized PLA-*g*-P(NIPAm-*co*-MAA) nanoparticles with core-shell structure were thremo-responsive, pH-responsive and biodegradable. The drug loading level of 5-FU encapsulated in this nanoparticles could reach 20% [32]. Dias et al. synthesized molecularly imprinted particles with surface grafted functional polymer brushes aiming at the targeting of 5-FU. For particles with PNIPA grafted brushes, a boost in drug release was also shown at 20 °C as compared to 40 °C [15,33]. In this work, the release behaviors of the 5-FU loaded NIPAM-MAPOSS hydrogels in PBS (pH 7.4) were evaluated at 37 °C for 9 h. The solubility of 5-FU in saline solution is about 1.45 mol/L at 37 °C [34]. All types of gels functioned as drug delivery systems that control the release of 5-FU.We selected several representative examples to investigate the effect of MAPOSS and PEG on the release efficiency and sustained release time of hydrogel. The cumulative release percentage of these groups of gels was listed in Table S1. The data for drug release (time from 1 to 9h) were fitted to a logarithmic function as follows:

(4) y=kln(x)+b,

where *k* affects the slope of the curve, *b* is directly related to the value of *y*, and *y* represents the cumulative release. The value of *k* is larger, the drug release is gentle. The release curves and the functions were shown in Figure 7.

The curves of Gel 1, Gel 3, and Gel 5 reflect the effect of MAPOSS on drug release performance. The final cumulative release of Gel 1 and Gel 3 are close, more than 70%. At the initial stage, the release rate of Gel 1 (without MAPOSS) was fast. Meanwhile, the cumulative release percentage of Gel 3 (containing 8.33 wt % MAPOSS) was lower than Gel 1, and then achieved a steady, sustained release in 300 min. Gel 3 had an ideal release rate and release efficiency. However, when the amount of MAPOSS continued to increase in the feed, the cumulative release percentage of Gel 5 became low. This was because the unique spatial structure of MAPOSS was conducive to the storage and slow release of drugs. MAPOSS also made the polymer network structure more solid, so as to achieve a sustained and stable release. However, too tight network structure limited the water in the hydrogel in and out, was against to drug loading and release. The results demonstrate that the addition of appropriate amount of MAPOSS can prolong the release time while maintaining the cumulative release percentage. Moreover, the curves of Gel 3, Gel 6, and Gel 8 show the effect of PEG on drug release performance of gels. The Gel 8 release curve was above any other curve. Nearly 76% of the 5-FU adsorbed in Gel 8 was released in 180 min, while Gel 6 only released 39% over the same time. And after 240 min, Gel 8 was barely released. The simulated curve of Gel 3 had a larger value of k than the others. The *b* value of the simulated release curve decreased as the amount of MAPOSS increases and increased with the amount of PEG. We can conclude that the release efficiency of macroporous hydrogel is high, but the duration of the sustained release is short.

## 4. Conclusions

A series of PNIPAM-MAPOSS hybrid hydrogels were prepared by one-step radical polymerization and utilized for drug delivery. From the morphology, the addition of MAPOSS made the internal pores heterogeneous and the network tight. Microphase-separation could be observed. The hydrophobic MAPOSS domains functioned as cross-linking sites, linking PNIPAM chains. The amount of PEG directly determined the size of pores. The PNIPAM-MAPOSS hybrid hydrogels were temperature sensitive because of NIPAM. However, adding MAPOSS decreased its LCST. The tight network structure and hydrophobicity reduced the *SR*s of gels. However, the hydrophobic domains could behave as the micro-porogens to facilitate the diffusion of water molecules, thereby increasing the temperature response rate of the hydrogels. The incorporation of rigid MAPOSS into the hydrogels greatly improved their compressive modulus. Just adding a small amount of MAPOSS could increase the compressive modulus of hydrogel approximately 10 times. This can be explained as MAPOSS increased the cross-linking degree of gels and made the network more robust. Additionally, the dispersion of POSS cages limited the movement of the polymer chains. The right amount of MAPOSS also helped to prolong the sustained release of drugs. MAPOSS was conducive to the storage and sustained release of drugs, and made the polymer network difficult to be destroyed in the water. PEG essentially affected the performance of hydrogels by changing the size of the pores. The large-pore hydrogel had a high swelling ratio, fast deswelling rate, and greater drug loading. However, its loaded drug released quickly and its mechanical properties might be weakened. More importantly, we obtained Gel 3 by adjusting the amount of MAPOSS and PEG, had a moderate pore size, excellent swelling behavior, and enhanced mechanical properties. It can serve as a drug carrier for 5-FU to treat cancer, but also for other biomedical applications.

## Figures and Tables

**Table 1:** PNIPAM-MAPOSS hybrid hydrogels composition and LCSTs.

Sample	Gels Formulation (g) ^1^		MAPOSS (wt %)	LCST (°C) ^2^
	MAPOSS	PEG		
Gel 1	0	0.10	0	31.1
Gel 2	0.05	0.10	4.35	31.1
Gel 3	0.10	0.10	8.33	31.1
Gel 4	0.20	0.10	15.38	29.4
Gel 5	0.30	0.10	21.43	28.5
Gel 6	0.10	0	8.33	31.1
Gel 7	0.10	0.05	8.33	31.1
Gel 8	0.10	0.30	8.33	31.1

^1^ The amount of raw material for preparing gels; ^2^ Defined by the endothermic peaks of the DSC curves.

## Supplementary Materials

The following are available online at http://www.mdpi.com/2073-4360/10/2/137/s1, Figure S1: The pore size distribution of PNIPAM-MAPOSS hybrid hydrogels: (a) Gel 6, (b) Gel 7, (c) Gel 3, and (d) Gel 8; Table S1: The cumulative release percentage (%) of Gel 1, Gel 3, Gel 5, Gel 6, and Gel 8.
