# Supplementary material for: PNIPAM-MAPOSS Hybrid Hydrogels with Excellent Swelling Behavior and Enhanced Mechanical Performance: Preparation and Drug Release of 5-Fluorouracil
Source: Polymers (Basel). 2018 Jan 31;10(2):137. doi: 10.3390/polym10020137 (PMC6414838; doi:10.3390/polym10020137)
Supplement: Supplementary file 1 [file polymers-10-00137-s001.pdf]

Supplementary Materials
for

PNIPAM-MAPOSS Hybrid Hydrogels with Excellent Swelling Behavior and Enhanced Mechanical Performance: Preparation and Drug Release of 5-Fluorouracil

Peihong Li, Xiaoman Hou, Lijie Qu, Xueyan Dai and Chunling Zhang *

In the SEM image, the pore size was measured manually. The pore size distribution of gels was shown in Figure S1.

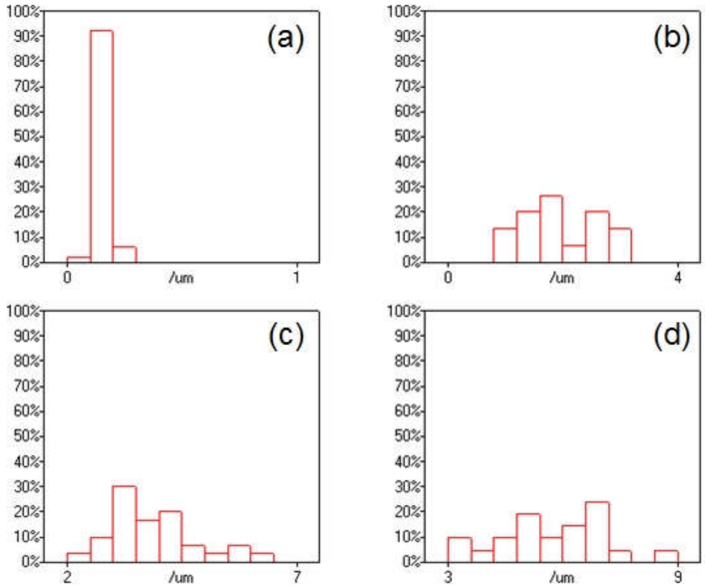

Figure S1. The pore size distribution of PNIPAM-MAPOSS hybrid hydrogels: (**a**) Gel 6, (**b**) Gel 7, (**c**) Gel 3, (**d**) Gel 8.

The release behaviors of the 5-FU loaded NIPAM-MAPOSS hydrogels in PBS (pH 7.4) were evaluated at 37 ℃. The cumulative release percentage of Gel 1, Gel 3, Gel 5, Gel 6 and Gel 8 over a period of 9 hours was listed in Table S1.

Table S1. The cumulative release percentage (%) of Gel 1, Gel 3, Gel 5, Gel 6 and Gel 8.

Time (h)	Gel 1	Gel 3	Gel 5	Gel 6	Gel 8
0.25	45.56	35.81	27.35	17.66	55.43
0.5	55.47	44.72	35.66	22.91	63.38
1	58.08	48.84	39.47	30.79	68.90
2	67.01	55.56	46.03	36.28	76.51
3	69.63	61.29	48.92	40.30	76.81
4	70.50	64.94	51.77	41.58	78.58
5	71.15	67.77	53.28	43.21	79.25
7	73.50	69.84	54.87	47.34	79.71
9	74.23	70.92	55.15	49.15	80.44
